# Electrocardiographic Changes with Age in Japanese Patients with Noonan Syndrome

**DOI:** 10.3390/jcdd11010010

**Published:** 2023-12-28

**Authors:** Yasuhiro Ichikawa, Hiroyuki Kuroda, Takeshi Ikegawa, Shun Kawai, Shin Ono, Ki-Sung Kim, Sadamitsu Yanagi, Kenji Kurosawa, Yoko Aoki, Mari Iwamoto, Hideaki Ueda

**Affiliations:** 1 Department of Cardiology, Kanagawa Children’s Medical Center, 2-138-4 Mutsukawa, Minami-ku, Yokohama 232-8555, Kanagawa, Japanikegawa.1120l@kanagawa-pho.jp (T.I.); kawai.shu.zf@yokohama-cu.ac.jp (S.K.); kskim9399@gmail.com (K.-S.K.);; 2 Department of Pediatric Cardiology, Yokohama City University Hospital, 3-9 Fukuura, Kanazawa-ku, Yokohama 236-0004, Kanagawa, Japan; 3 Division of Medical Genetics, Kanagawa Children’s Medical Center, 2-138-4 Mutsukawa, Minami-ku, Yokohama 232-8555, Kanagawa, Japan; 4 Department of Medical Genetics, Tohoku University Graduate School of Medicine, 1-1 Seiryo-machi, Aoba-ku, Sendai 980-8574, Miyagi, Japan; 5 Department of Pediatrics, Saiseikai Yokohama-shi Tobu Hospital, 3-6-1 Shimosueyoshi, Tsurumi-ku, Yokohama 230-8765, Kanagawa, Japan

**Keywords:** Noonan syndrome, electrocardiogram, right-axis deviation, small R, T-positive V1

## Abstract

Little information is available on age-related electrocardiographic changes in patients with Noonan syndrome. This single-center study evaluated the electrocardiograms of patients with Noonan syndrome. We divided the patients (*n* = 112; electrocardiograms, 256) into four groups according to age: G1 (1 month–1 year), G2 (1–6 years), G3 (6–12 years), and G4 (>12 years). Typical Noonan syndrome-related electrocardiographic features such as left-axis deviation, abnormal Q wave, wide QRS complex, and small R wave in precordial leads were detected. A high percentage of QRS axis abnormalities was found in all groups. Significant differences in right-axis deviation (RAD) were noted among the groups: 56.5% of G1 patients showed RAD compared with 33.3% of G2, 21.1% of G3, and 19.2% of G4 patients. The small R was also significantly different among the groups: 32.6% of G1 patients showed a small R wave compared with 14.9% of G2, 8.5% of G3, and 15.4% of G4 patients. Of the 53 patients with Noonan syndrome aged 1 month to 2 years, 18 had T-positive V1 with a higher prevalence of pulmonary stenosis and cardiac interventions. QRS axis abnormalities, small R in V6, and T-positive V1 could help diagnose Noonan syndrome in infants or young children.

## 1. Introduction

Noonan syndrome is an autosomal dominant disorder characterized by short stature, craniofacial dysmorphism, congenital heart disease, skeletal abnormalities, developmental delay, hematological disorders, and other abnormalities [1]. The estimated incidence is 1:1000 to 1:2500 live births [2]. Noonan syndrome is caused by genetic mutations related to the RAS–mitogen-activated protein kinase signal transduction pathway [3]. Mutations in the following genes that cause Noonan syndrome have been identified: RAS family GTPase proteins (*KRAS*, *NRAS*, *RIT1*, and *RRAS*), RAS signal function modulators (*PTPN11*, *SOS1*, *SOS2*, *CBL*, *RASA2*, and *SHOCS2*), and downstream signal transducers (*RAF1* and *BRAF*) [3].

Heart disease is one of the most important complications of Noonan syndrome. The prevalence of cardiovascular disease in patients with Noonan syndrome has been reported to be 82–90% [4]. The most common cardiovascular diseases are pulmonary stenosis (PS), hypertrophic cardiomyopathy (HCM), and atrial septal defect (ASD) [2]. Some patients with severe PS require treatment that affects their quality of life. HCM, detected in 20% of patients with Noonan syndrome, reduces patient survival [5].

**Figure 1:**
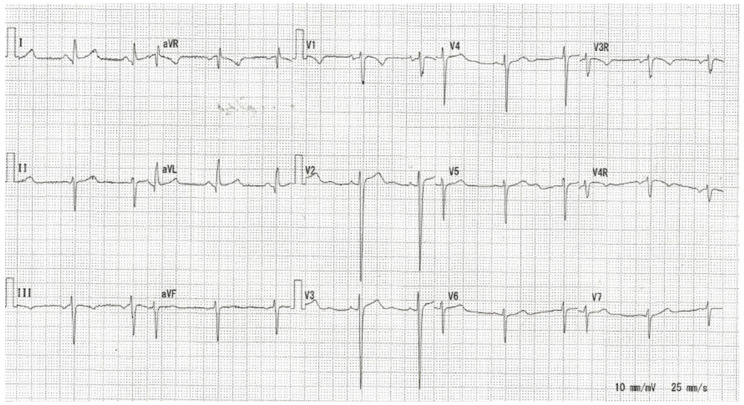
Electrocardiogram (speed 25 mm/s, voltage 0.1 mV/mm) of a 10-year-old patient with Noonan syndrome with pulmonary stenosis and atrial septal defect.

Electrocardiograms of patients with Noonan syndrome exhibit typical findings [6,7,8,9,10] such as left-axis deviation (45–61%), small R waves in the left precordial leads (20–31%), wide QRS complex (0–28%), and abnormal Q wave (2–43%) [6,7,8,9]. Figure 1 shows an electrocardiogram of a patient with Noonan syndrome. Previous reports indicated that these typical electrocardiogram findings in Noonan syndrome were not associated with *PTPN11* mutations and were not related to congenital heart disease [8,9]. However, the exact mechanism underlying the typical electrocardiographic features of Noonan syndrome is not well understood [7].

Recently, genetic analysis has made it possible to diagnose Noonan syndrome; however, it is not always possible to do this in younger children. In these cases, cardiac findings, including electrocardiographic findings, are helpful in diagnosing Noonan syndrome. As electrocardiographic findings vary with age in children, information on age-related electrocardiogram changes in Noonan syndrome is lacking, and additional research is required. Additionally, previous reports have focused on the typical electrocardiographic findings of Noonan syndrome, and little is known about other diagnostic electrocardiographic characteristics [6,7,8,9,10]. In the present study, we investigated age-related electrocardiographic changes in pediatric patients with Noonan syndrome.

## 2. Materials and Methods

### 2.1. Patients

We retrospectively reviewed the medical records of the Kanagawa Children’s Medical Center. Patients who were clinically or genetically diagnosed with Noonan syndrome by medical geneticists since the hospital opened (April 1970) to December 2021 were included. We used the clinical diagnostic criteria of van der Burgt [11], especially for cases in which there was no molecular diagnosis. For cases before these diagnostic criteria were proposed, clinical evaluations by the clinical geneticists were used in the scoring system to examine the correctness of the clinical diagnosis. The inclusion criteria required that patients should undergo at least one evaluation that included an electrocardiogram. Patients with suspected cardiofacio–cutaneous syndrome or Costello syndrome were excluded. Clinical characteristics, including age, sex, body weight, height, type of cardiovascular anomaly, echocardiographic data, information about cardiac surgery, and responsible mutations, were collected from the medical records. The study was approved by the Ethics Committees of Kanagawa Children’s Medical Center (approval no. 2101-7) and Tohoku University School of Medicine (approval nos. 2015-1-222, 2021-1-271).

### 2.2. Electrocardiogram

The specific electrocardiographic findings in patients with Noonan syndrome reported in previous studies and included in this study were left-axis deviation, small R waves in the left precordial leads, abnormal Q waves, and/or wide QRS complexes [6,7,9]. We also assessed other electrocardiographic findings such as right-axis deviation and bundle branch block. Right-axis deviation was defined as a QRS axis above the upper normal limit for the patient’s age [12]. Left-axis deviation was defined as a QRS axis below the lower normal limit for the patient’s age [12]. The northwest axis was included in the left-axis deviation. The age-specific normal ranges of the QRS axis (degree) are as follows: 30–115 (1–3 months), 7–105 (4–6 months), 6–98 (7–12 months), 7–102 (1–3 years), 6–104 (3–5 years), 10–139 (5–8 years), 6–116 (8–12 years), and 9–128 (12–16 years). Small R waves in V6 were defined as little R deflections over the left precordium with an R/S ratio below the lowest normal limits and an R voltage in V5 and V6, which was less than 50% of the mean, as described by Park and Guntheroth [13,14]. An abnormal Q wave was defined as a Q voltage above the upper normal limit and longer than 0.04 s [6]. A wide QRS complex was defined as a QRS duration >0.08 s for patients <3 years of age, >0.10 s for patients from 3–12 years of age, and above the upper limit of 0.12 s for patients >12 years of age [6,14].

The electrocardiograms were divided into four groups based on the patient age: group 1 (1 month–1 year), group 2 (1–6 years), group 3 (6–12 years), and group 4 (>12 years). For the analysis of the T wave in V1, electrocardiograms of patients aged 1 month–2 years were used. One electrocardiogram per patient was evaluated for each group. When multiple electrocardiograms for one patient in each group were available, the electrocardiogram of the patient whose age was closest to the median age of the group was used.

### 2.3. Mutation Analysis

Genomic DNA was extracted from peripheral blood samples obtained from the patients and their family members using standard procedures [15]. Mutation screening was performed by direct sequencing of exons and their flanking regions in the responsible genes, including *PTPN11*, *KRAS*, *BRAF*, *MAP2K1/2*, and *HRAS* [16]. We used either a targeted next-generation sequencing panel of 41 genes responsible for Noonan syndrome and related diseases, the TruSight One Sequencing Panel (Illumina, Inc., San Diego, CA, USA), or whole-exome sequencing [17,18]. Variants identified by targeted sequencing were confirmed by Sanger sequencing.

### 2.4. Statistical Analysis

Data are presented as the mean ± standard error of the mean for independent experiments. The four groups were compared statistically, and differences were identified using one-way analysis of variance or Fisher’s exact test. Differences between two groups were identified using an unpaired two-tailed Student’s *t*-test or Fisher’s exact test. Statistical significance was set at *p* < 0.05.

## 3. Results

A total of 112 patients (256 electrocardiograms) with Noonan syndrome met the inclusion criteria at our institution. To investigate the electrocardiographic changes with age, we divided the electrocardiograms into four groups based on patient age. Before analyzing the electrocardiograms of each group, the medical histories of the patients in the four groups were extracted from the medical records. Table 1 shows the demographic characteristics of the patients in the four groups. There were no significant differences among the four groups in the overall prevalence of congenital heart diseases and cardiovascular diseases such as PS, HCM, and ASD. Thirty-nine patients had genetic mutations responsible for Noonan syndrome. The remaining patients did not undergo genetic testing and were diagnosed based on clinical findings. The genetic mutations included the following: *PTPN11* (24 patients), *SOS1* (six patients), *RIT1* (four patients), *KRAS* (two patients), *RAF1* (one patient), *BRAF* (one patient), and *SHOCS2* (one patient).

Thirty-four patients underwent cardiac intervention. Twenty-one of them underwent both preoperative and postoperative electrocardiography. Table S1 shows the electrocardiographic data before and after the cardiac intervention. No other significant differences were found between the preoperative and postoperative groups.

**Figure 2:**
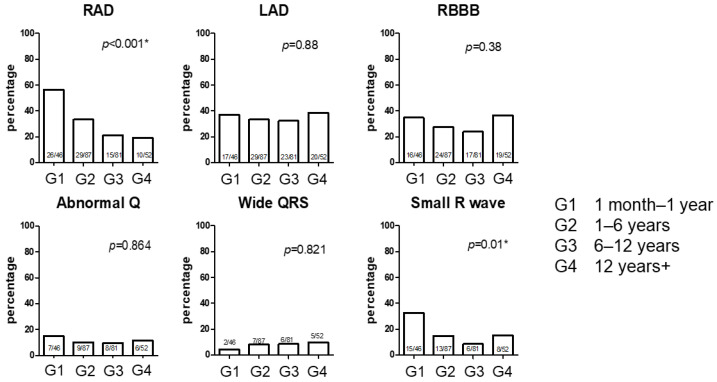
Percentage of electrocardiogram findings by age group. RAD, right-axis deviation; LAD, left-axis deviation; RBBB, right bundle branch block; abnormal Q, abnormal Q wave; Small R wave, small R wave in V6. * *p* < 0.05 was considered significant.

We then analyzed the electrocardiographic features of the four groups (Table 2, Figure 2). Similar to previous studies, typical features of Noonan syndrome-related electrocardiographic features were observed. These included left-axis deviation (32.4–38.4%), abnormal Q waves (10.3–15.2%), wide QRS complexes (4.3–9.6%), and small R waves (8.5–32.6%). Most patients exhibited QRS axis abnormalities (left- or right-axis deviation). There was a significant difference in right-axis deviation among the four groups. Right bundle branch block was detected in approximately 30% of patients in all four groups. One patient exhibited a left bundle branch block, underwent resection of the left ventricular outflow tract with severe HCM, and showed a left bundle branch block after surgery. A wide QRS complex was detected in all groups, and the presence of small R waves was significantly different among the four groups. The R/S ratios in V6 and RV6 were also significantly different among the four groups.

More than 50% and more than 30% of infants with Noonan syndrome were observed to have right-axis deviation and small R waves, respectively. Therefore, we analyzed the phenotypic differences in the cardiovascular system with or without each electrocardiographic finding in group 1 (Table 3). The prevalence of cardiovascular phenotypes was not significantly different between the two groups for left- and right-axis deviations, right bundle branch block, abnormal Q waves, and wide QRS complexes. HCM was more prevalent in patients with small R waves. We also compared the genetically diagnosed group (*PTPN11* 7, *SOS1* 3, *RIT1* 3, *RAF1* 1, *KRAS* 1) and the clinically diagnosed group in group 1 and found no significant difference in the incidence of electrocardiographic findings characteristic of Noonan syndrome (Table S2). 

Additionally, we examined the percentage of V1 T-positive patients from 1 month to 2 years of age (Table 4). Eighteen of the 53 patients (33.9%) were T-positive in V1. The prevalence of PS and the percentage of cardiac intervention (surgery or catheter intervention) in patients with T-positive V1 were significantly higher than those in patients with T-negative V1. 

## 4. Discussion

The present cohort of Japanese patients provides new insights into the electrographic diagnosis of Noonan syndrome. As in previous reports, the typical electrocardiographic findings of patients with Noonan syndrome, such as left-axis deviation, small R waves in the left precordial leads, a wide QRS complex, and abnormal Q waves, were observed in all age groups [6,7,8,9]. Additionally, right-axis deviation and left precordial small R waves showed significant differences among the four groups. Furthermore, a high prevalence of Noonan syndrome with T-positive V1 was observed in younger children and was associated with the prevalence of PS and cardiac interventions. 

An electrocardiogram is a useful tool for evaluating cardiovascular diseases in children and may provide a basis for cardiac screening [19]. In the present study, we focused on the QRS axis. Although left-axis deviation in infants and children has been associated with structural heart diseases such as tricuspid atresia, atrioventricular septal defect, and corrected transposition of the great arteries, a previous study indicated that 1% of healthy children have left-axis deviation [20]. Compared with healthy children, approximately 40% of patients with Noonan syndrome in our cohort exhibited left QRS axis deviation in any age group. Therefore, left-axis deviation may help in the diagnosis of Noonan syndrome at any age. Right-axis deviation was not emphasized as a specific feature of Noonan syndrome in previous studies [6,7,8,9,10]. In our study, right-axis deviation showed significant differences among the four groups, and more than 50% of the infants with Noonan syndrome had right-axis deviation. Right-axis deviation may reflect physiological changes in right ventricular hypertrophy during the neonatal period. Although the presence of right-axis deviation could be expected based on the hemodynamics of heart diseases such as PS and ASD, no association was found between right-axis deviation and congenital heart disease in our Noonan cohort [21]. One explanation for this is that the severity of cardiovascular disease may differ among patients. Most of the infants had QRS axis abnormalities, whether left- or right-axis deviations. We believe that QRS axis abnormalities, including both left- and right-axis deviations, may be characteristic electrocardiographic findings in infants with Noonan syndrome.

The small R wave in V6 was also significantly different among the four groups, and 32.6% of infants with Noonan syndrome showed small R waves in V6. We also believe that small R waves in V6 may be characteristic of electrocardiographic findings in infants with Noonan syndrome. A small R wave consists of an R-to-S wave voltage ratio. In the present cohort, the RV6 voltage was significantly different among the four groups, but there was no significant difference in SV6. The significant difference in the small R waves at V6 was caused by a change in RV6. The R-wave voltage was also age-dependent [12]. 

Previous studies have reported that approximately 1% of healthy children have a right bundle branch block [22]. A right bundle branch block was more frequently detected in our cohort than in the healthy children group, although this was not emphasized in previous studies. Right bundle branch block, in addition to the characteristic electrocardiographic findings, could also be useful in diagnosing Noonan syndrome at any age.

T-positive V1 is an electrocardiographic finding for right ventricular hypertrophy in infants and younger children [23]. Patients with PS exhibit right ventricular hypertrophy due to high right ventricular pressure [24]. Although previous reports have indicated that typical electrocardiographic findings in Noonan syndrome are not associated with congenital heart disease, our results showed that a positive T wave in V1 was related to the prevalence of PS and cardiac intervention in younger pediatric patients with Noonan syndrome [7]. 

This study had some limitations. Because all examinations, including electrocardiography, were performed in clinical settings, some electrocardiogram and echocardiogram data were missing and not all information was available for each age group. Additionally, several patients with Noonan syndrome underwent electrocardiography and echocardiography in the 1980s, and their echocardiographic data such as pulmonary flow velocity could not be accessed. Therefore, we could not link electrocardiographic findings with cardiovascular disease severity in this cohort. 

Based on this study, we believe that common electrocardiographic findings in infants with Noonan syndrome are QRS axis abnormalities including left- and right-axis deviations and T positivity in V1, in addition to the characteristic findings (left-axis deviation, small R waves in the left precordial leads, wide QRS complex, and abnormal Q waves) reported previously. In conclusion, right-axis deviation, small R in V6, and T positivity in V1, as well as typical electrocardiographic features, could help diagnose Noonan syndrome in infants or young children.

## Figures and Tables

**Table 1:** Cardiovascular disease in patients with Noonan syndrome.

Group	1 (1 Month–1 Year)	2 (1–6 Years)	3 (6–12 Years)	4 (>12 Years)	*p*	Total
n	46	87	71	52		112
Age (years) (SD)	0.42 (0.26)	4.04 (1.29)	8.76 (1.72)	15.35 (3.34)		
Sex (men) (%)	25 (54.3)	48 (55.1)	38 (53.5)	30 (57.6)		67 (59.8)
HCM (%)	14 (30.4)	20 (23.0)	17 (23.9)	15 (28.8)	0.732	22 (19.6)
PS (%)	29 (63.0)	44 (50.6)	35 (40.3)	25 (48.1)	0.417	53 (47.3)
ASD (%)	15 (32.6)	21 (24.1)	16 (22.5)	12 (23.1)	0.632	28 (25.0)
VSD (%)	3 (6.5)	4 (4.6)	5 (7.0)	4 (7.7)	0.86	6 (5.3)
CoA (%)	0 (0.0)	1 (1.1)	1 (1.4)	0 (0.0)	0.458	1 (0.9)
TOF (%)	1 (2.2)	1 (1.1)	1 (1.4)	1 (1.9)	1	1 (0.9)
AVSD (%)	1 (2.2)	1 (1.1)	1 (1.4)	1 (1.9)	1	1 (0.9)
PDA (%)	2 (4.3)	2 (2.3)	2 (2.8)	0 (0.0)	0.521	2 (1.8)
Coronary stenosis (%)	1 (2.2)	1 (1.1)	1 (1.4)	1 (1.9)	1	2 (1.8)
AR (%)	0 (0.0)	0 (0.0)	0 (0.0)	1 (1.9)	0.382	1 (0.9)
Total CHD (%)	41 (89.1)	66 (75.8)	53 (74.6)	42 (80.8)	0.38	81 (72.3)

HCM, hypertrophic cardiomyopathy; PS, pulmonary stenosis; ASD, atrial septal defect; VSD, ventricular septal defect; CoA, coarctation of the aorta; TOF, tetralogy of Fallot; AVSD, atrioventricular septal defect; PDA, patent ductus arteriosus; AR, aortic regurgitation, CHD; congenital heart disease; SD, standard deviation.

**Table 2:** Electrocardiogram abnormalities in patients with Noonan syndrome.

Group	1 (1 Month−1 Year)	2 (1−6 Years)	3 (6−12 Years)	4 (>12 Years)	*p*
n	46	87	71	52	
HR (beats/min)	140.00 (15.63)	107.38 (22.06)	82.14 (15.58)	76.21 (15.38)	<0.001
PR (ms)	0.12 (0.01)	0.14 (0.02)	0.14 (0.02)	0.15 (0.03)	<0.001
QT (ms)	0.28 (0.02)	0.32 (0.04)	0.37 (0.04)	0.39 (0.03)	<0.001
QTcB (ms)	0.43 (0.03)	0.43 (0.03)	0.43 (0.03)	0.43 (0.03)	0.315
QTcF (ms)	0.38 (0.02)	0.39 (0.03)	0.41 (0.03)	0.42 (0.02)	<0.001
QRS (ms)	0.10 (0.13)	0.08 (0.01)	0.09 (0.01)	0.11 (0.11)	0.151
Axis (degree)	51.02 (123.88)	46.18 (99.71)	35.92 (81.63)	30.75 (86.04)	0.682
R/S V6	1.54 (3.02)	3.27 (4.82)	4.78 (4.95)	3.74 (3.83)	0.002
SV1 (mV)	0.96 (0.84)	1.13 (1.13)	1.32 (1.29)	1.05 (0.91)	0.32
RV1 (mV)	1.09 (0.63)	0.70 (0.58)	0.60 (0.54)	0.66 (0.82)	<0.001
SV6 (mV)	0.76 (0.48)	0.56 (0.60)	0.46 (0.71)	0.68 (1.06)	0.119
RV6 (mV)	0.57 (0.43)	0.76 (0.68)	0.89 (0.64)	0.90 (0.58)	0.02
Q in V6 (mV)	0.04 (0.09)	0.09 (0.44)	0.11 (0.60)	0.16 (0.79)	0.745
Q in AVF (mV)	0.07 (0.13)	0.06 (0.16)	0.04 (0.10)	0.07 (0.20)	0.472
Abnormal Q (%)	7 (15.2)	9 (10.3)	8 (11.3)	6 (11.5)	0.864
ST elevation (%)	0 (0.0)	3 (3.4)	2 (2.8)	2 (3.8)	0.71
ST depression (%)	1 (2.2)	4 (4.6)	5 (7.0)	2 (3.8)	0.733
Negative T (%)	1 (2.2)	4 (4.6)	6 (8.5)	1 (1.9)	0.365
AV block (%)	0 (0.0)	0 (0.0)	0 (0.0)	2 (3.8)	0.072
PAC (%)	1 (2.2)	2 (2.3)	1 (1.4)	0 (0.0)	0.836
PVC (%)	0 (0.0)	0 (0.0)	0 (0.0)	1 (1.9)	0.383

HR, heart rate; PR, PR interval; QT, QT interval; QTcB, QT interval corrected using Bazett’s formula; QTcF, QT interval corrected using Fridericia’s formula; QRS, QRS interval; Axis, QRS axis; abnormal Q, abnormal Q wave; negative T, negative T wave; AV block, atrioventricular block; PAC, premature atrial contraction; PVC, premature ventricular contraction.

**Table 3:** Cardiovascular disease in infants with Noonan syndrome with and without specific electrocardiogram features (LAD, RAD, RBBB, abnormal Q wave, and wide QRS complex).

	LAD; Group 1			RAD; Group 1			RBBB; Group 1		
	Positive	Negative	*p*	Positive	Negative	*p*	Positive	Negative	*p*
N of patient	17	29		26	20		16	30	
HCM	6	8	0.741	7	7	0.077	3	11	0.812
ASD	9	6	0.471	6	9	0.203	6	9	0.203
PS	10	19	0.411	18	11	0.368	10	19	1
Total CHD	15	26	1	24	17	0.639	13	28	0.324
Total intervention	6	12	0.647	12	6	0.364	8	10	0.347
	Small R; Group 1			Abnormal Q; Group 1			Wide QRS; Group 1		
	positive	negative	*p*	positive	Negative	*p*	positive	negative	*p*
N of patient	15	31		7	39		2	44	
HCM	8	6	0.0377	2	12	1	1	13	-
ASD	5	10	1	0	15	0.0782	0	15	-
PS	9	20	1	4	25	1	1	28	-
Total CHD	14	27	1	7	32	0.572	2	39	-
Total intervention	8	10	0.208	2	16	1	2	16	-

HCM, hypertrophic cardiomyopathy; PS, pulmonary stenosis; ASD, atrial septal defect; CHD; congenital heart disease; LAD, left-axis deviation; RAD, right-axis deviation; RBBB, right bundle branch block.

**Table 4:** Cardiovascular diseases in patients with Noonan syndrome aged 1 month–2 years with and without T-positive V1.

	T Wave in V1		
	Positive	Negative	*p*
N of patient	18	35	
HCM	6	10	0.667
ASD	2	9	0.295
PS	15	16	0.0098
Total CHD	17	29	0.651
Total intervention	13	6	0.0002

HCM, hypertrophic cardiomyopathy; PS, pulmonary stenosis; ASD, atrial septal defect; CHD, congenital heart disease.

## Data Availability

Data is available on request from the authors.

## Supplementary Materials

The following supporting information can be downloaded at: https://www.mdpi.com/article/10.3390/jcdd11010010/s1, Table S1: Pre- and postoperative electrocardiogram abnormalities in patients with Noonan syndrome, Table S2: Electrocardiographic changes in Noonan syndrome with genetic and clinical diagnosis.
