# Supplementary material for: Electrocardiographic Changes with Age in Japanese Patients with Noonan Syndrome
Source: J Cardiovasc Dev Dis. 2023 Dec 28;11(1):10. doi: 10.3390/jcdd11010010 (PMC10816141; doi:10.3390/jcdd11010010)
Supplement: Supplementary file 1 [file jcdd-11-00010-s001.zip › jcdd-2689539-supplementary.pdf]

Table S1

Group	Pre-surgery	Post-surgery	*p*
n	21	21	
RAD (%)	10 (47.6)	9 (42.8)	1
LAD (%)	9 (42.8)	5 (23.8)	0.326
RBBB (%)	10 (47.6)	12 (57.1)	0.523
LBBB (%)	0 (0.0)	1 (4.7)	1
LVH (%)	1 (4.7)	1 (4.7)	1
RVH (%)	3 (14.2)	1 (4.7)	0.606
Abnormal Q (%)	3 (14.2)	4 (19.0)	1
ST elevation (%)	0 (0.0)	1 (4.7)	1
ST depression (%)	0 (0.0)	1 (4.7)	1
negative T (%)	1 (4.7)	1 (4.7)	1
AV block (%)	0 (0.0)	0 (0.0)	1
PAC (%)	0 (0.0)	0 (0.0)	1
PVC (%)	0 (0.0)	0 (0.0)	1
wide QRS (%)	3 (14.2)	5 (23.8)	0.696
Small R (%)	5 (23.8)	6 (28.5)	1

RAD, right-axis deviation; LAD, left-axis deviation; RBBB, right bundle branch block; LBBB, left bundle branch block; LVH, left ventricular hypertrophy; RVH, right ventricular hypertrophy; abnormal Q, abnormal Q wave; negative T, negative T wave; AV block, atrioventricular block; PAC, premature atrial contraction; PVC, premature ventricular contraction; Small R wave, small R wave in V6.

Table S2

	genetic diagnosis (n=15)	clinical diagnosis (n=31)	*p*
RAD	8/15 (53.3)	18/31 (58.1)	>0.99
LAD	6/15 (40.0)	11/31 (35.4)	>0.99
RBBB	7/15 (46.6)	9/31 (29.0)	0.32
Abnormal Q	1/15 (6.7)	6/31 (19.3)	0.39
Wide QRS	0/15 (0.0)	2/31 (6.4)	>0.99
Small R wave	4/15 (26.6)	11/31 (35.4)	0.73

RAD, right-axis deviation; LAD, left-axis deviation; RBBB, right bundle branch block; abnormal Q, abnormal Q wave; negative T, negative T wave; Small R wave, small R wave in V6.
